# Mutational and clinical spectrum of Japanese patients with hereditary hemorrhagic telangiectasia

**DOI:** 10.1186/s12920-021-01139-y

**Published:** 2021-12-06

**Authors:** Kana Kitayama, Tomoya Ishiguro, Masaki Komiyama, Takayuki Morisaki, Hiroko Morisaki, Gaku Minase, Kohei Hamanaka, Satoko Miyatake, Naomichi Matsumoto, Masaru Kato, Toru Takahashi, Tohru Yorifuji

**Affiliations:** 1 Division of Pediatric Endocrinology and Metabolism, Children’s Medical Center, Osaka City General Hospital, 2-13-22 Miyakojima-hondori, Miyakojima, Osaka, 534-0021 Japan; 2 Department of Neuro-Intervention, Osaka City General Hospital, Osaka, Japan; 3 Division of Molecular Pathology, The Institute of Medical Science, The University of Tokyo, Tokyo, Japan; 4 Department of Bioscience and Genetics, National Cerebral and Cardiovascular Center, Osaka, Japan; 5 Department of Medical Genetics, Sakakibara Heart Institute, Tokyo, Japan; 6 Department of Human Genetics, Yokohama City University Graduate School of Medicine, Kanagawa, Japan; 7 Department of Genetic Medicine, Osaka City General Hospital, Osaka, Japan

**Keywords:** *ENG*, *ACVRL1*, Hereditary hemorrhagic telangiectasia

## Abstract

**Background:**

Hereditary hemorrhagic telangiectasia (HHT) is a dominantly inherited vascular disorder characterized by recurrent epistaxis, skin/mucocutaneous telangiectasia, and organ/visceral arteriovenous malformations (AVM). HHT is mostly caused by mutations either in the *ENG* or *ACVRL1* genes, and there are regional differences in the breakdown of causative genes. The clinical presentation is also variable between populations suggesting the influence of environmental or genetic backgrounds. In this study, we report the largest series of mutational and clinical analyses for East Asians.

**Methods:**

Using DNAs derived from peripheral blood leukocytes of 281 Japanese HHT patients from 150 families, all exons and exon–intron boundaries of the *ENG*, *ACVRL1*, and *SMAD4* genes were sequenced either by Sanger sequencing or by the next-generation sequencing. Deletions/amplifications were analyzed by the multiplex ligation-dependent probe amplification analyses. Clinical information was obtained by chart review.

**Results:**

In total, 80 and 59 pathogenic/likely pathogenic variants were identified in the *ENG* and *ACVRL1* genes, respectively. No pathogenic variants were identified in the *SMAD4* gene. In the *ENG* gene, the majority (60/80) of the pathogenic variants were private mutations unique to a single family, and the variants were widely distributed without any distinct hot spots. In the *ACVRL1* gene, the variants were more commonly found in exons 5–10 which encompasses the serine/threonine kinase domain. Of these, 25/59 variants were unique to a single family while those in exons 8–10 tended to be shared by multiple (2–7) families. Pulmonary and cerebral AVMs were more commonly found in *ENG*-HHT (69.1 vs. 14.4%, 34.0 vs. 5.2%) while hepatic AVM was more common in *ACVRL1*-HHT (31.5 vs. 73.2%). Notable differences include an increased incidence of cerebral (34.0% in *ENG*-HHT and 5.2% in *ACVRL1*-HHT), spinal (2.5% in *ENG*-HHT and 1.0% in ACVL1-HHT), and gastric AVM (13.0% in *ENG*-HHT, 26.8% in *ACVRL1*-HHT) in our cohort. Intrafamilial phenotypic heterogeneity not related to the age of examination was observed in 71.4% and 24.1% of *ENG*- and *ACVRL1*-HHT, respectively.

**Conclusions:**

In a large Japanese cohort, *ENG*-HHT was 1.35 times more common than *ACVRL1*-HHT. The phenotypic presentations were similar to the previous reports although the cerebral, spinal, and gastric AVMs were more common.

**Supplementary Information:**

The online version contains supplementary material available at 10.1186/s12920-021-01139-y.

## Background

Hereditary hemorrhagic telangiectasia (HHT), also known as Osler–Weber–Rendu syndrome, is a dominantly-inherited genetic disorder affecting 1 in 5–8000 individuals [1–4]. HHT is characterized by spontaneous epistaxis, multiple mucocutaneous telangiectasias, and arteriovenous shunts in various organs. The clinical diagnosis of HHT is made based on the Curaçao criteria [5]: (1) spontaneous, recurrent epistaxis; (2) multiple mucocutaneous telangiectasias at characteristic sites (lips, oral cavity, fingers, and nose); (3) visceral involvement including pulmonary, cerebral, hepatic, or gastrointestinal (GI) arteriovenous shunts; and (4) the presence of a first-degree relative with HHT. The diagnosis of HHT is ‘definite’ when > 3 criteria are met, ‘possible’ when 2 criteria are met, and ‘unlikely’ when < 2 criteria are met [5].

The majority (85–95%) of HHT patients have mutations either in the endoglin (*ENG*) gene (HHT1) or in the activin receptor-like kinase-1 (*ACVRL1*) gene (HHT2) [6–8]. Additionally, mutations in the SMAD family member 4 (*SMAD4*) gene cause a combined syndrome of HHT and juvenile polyposis [9] accounting for ~ 2% of the HHT cases and, recently, mutations in the GDF2 gene on chromosome 10, coding for bone morphogenetic protein 9 (BMP9), has been implicated as a cause of HHT (HHT5) [8]. These genes are all part of the bone morphogenetic protein (BMP)/transforming growth factor (TGFβ) signaling pathways and integral to angiogenesis [8] Pathogenic variants in any of these genes may disrupt the balance between pro- and antiangiogenic signals for normal vascular development, resulting in HHT. Other genetic loci have also been implicated by linkage analyses to two distinct loci on chromosomes 5 (HHT3) and 7 (HHT4) although no causative genes have been identified [10, 11].

So far, more than 1000 different mutations have been identified as associated with *ENG*-HHT and *ACVRL1*-HHT [12, 13]. However, most of the large-scale studies have been conducted on Caucasians [1, 14–16], and only limited, relatively small studies have been reported for other populations [17–19]. Moreover, even within Caucasians, the reported mutational spectrum has been different depending on the countries so does the clinical presentation [14–16, 20].

In this study, we report the results of the largest mutational analyses for Asian HHT patients together with the detailed clinical presentation.

## Subjects and methods

### Subjects

The study subjects were 281 (140 males, 141 females) Japanese patients from 150 families who were referred to Osaka City General Hospital during September 2010—June 2021, diagnosed with HHT, and underwent mutational analysis. Osaka City General Hospital is the largest HHT center in Japan with patients referred from across Japan.

### Methods

#### Clinical diagnosis

When patients with suspected HHT were referred to our institution, they routinely underwent the following diagnostic procedures: detailed past and family history taking, measurement of oxygen saturation, inspection for mucocutaneous telangiectasia, complete blood counts with differentials, blood chemistry, and electrolytes, followed by the diagnostic imaging including the brain, pulmonary, and liver vascular malformations. Specifically, for the brain imaging, non-contrast and contrast MR imaging including T1-, T2-, T2*-weighted, FLAIR, images and contrast-enhanced MR angiography at 1.5 or 3.0-T were performed. Additional routine imaging studies include dynamic, contrast-enhanced CT imaging of the liver (plain, early, and delayed phases), and axial contrast-enhanced images of the lung, spine, abdomen, and pelvis. The GI tract examination by the upper and lower tract fiberscope was performed only when severe anemia cannot be explained by the present epistaxis. When bleeding from the small intestine was suspected, capsule endoscopy was indicated.

Following these procedures, the probands from each family were diagnosed with definite/probable HHT by the Curaçao diagnostic criteria [5].

(Mutational analysis) Mutational analysis was proposed basically to all proband patients with the clinical diagnosis of HHT. Mutational analyses were also offered for selected family members including minors yet without a clinical diagnosis of HHT.

Using DNAs extracted from the peripheral blood leukocytes, all exons and exon–intron boundaries of the *ENG*, *ACVR1*, and *SMAD4* genes were sequenced either by Sanger sequencing using the ABI PRISM3130xl Genetic Analyzer (ThermoFisher, Waltham, USA) or by next-generation sequencing using the Ion PGM system (ThermoFisher), HiSeq 2000, or HiSeq 2500 (Illumina, San Diego, USA). In addition, multiplex ligation-dependent probe amplification (MLPA) analyses were performed using the probemix P093 HHT/HPAH (MRC Holland, Amsterdam, Netherlands) to detect deletion/amplification in the *ENG* and *ACVR1* genes. The pathogenic significance of the identified variants was determined according to the criteria of the American College of Medical Genetics [21]. The SIFT (https://sift.bii.a-star.edu.sg/), Polyphen 2 (http://genetics.bwh.harvard.edu/pph2/), and Combined Annotation-Dependent Depletion (CADD, https://cadd.gs.washington.edu/) programs were used for the determination of the pathogenicity scores. The clinical data were obtained by chart review.

The study protocol was approved by the Institutional Review Board at Osaka City General Hospital (No. 170850) and the study was performed under written informed consent from the patients or the guardians when appropriate.

## Results

Detailed mutational and clinical data of all patients are listed in supplemental Table 1.

**Table 1:** Breakdown of identified mutations of the probands

	*ENG*	*ACVRL1*	*SMAD4*	Not identified
Number of probands with pathogenic variants	80	59	0	11
Sex (male/female)	46/34	23/36	0	4/7
Novel variants	20	9	0	
De novo variants	3 (3.8%)	1 (1.7%)		
Variant types				
Missense	23 (28.8%)	38 (64.4%)	0 (0%)	
Nonsense/frameshift	28 (35.0%)	18 (30.5%)	0 (0%)	
Splice site	20 (25.0%)	3 (5.1%)	0 (0%)	
Large deletion	5 (6.3%)	0 (0%)	0 (0%)	
In-frame deletion of 12–18 bp	3 (3.8%)			
5'-UTR	1 (1.3%)			

*ENG* endoglin, *ACVRL1* activin A receptor like type 1, *SMAD4* SMAD Family Member 4, *UTR* untranslated region

Table 1 shows the identified pathogenic (P)/likely pathogenic (LP) variants of the probands from each family. In total, 80 and 59 P/LP variants were identified in the *ENG* and *ACVRL1* genes, respectively. No pathogenic variants were identified in the *SMAD4* gene. Eleven probands with the diagnosis of definite/possible HHT by the Curaçao diagnostic criteria did not have any P/LP variants. However, two of them were found to have 5’- untranslated region (UTR) variants in the *ENG* gene of unknown significance, −79C > T and −33A > G, respectively. Among the 80 P/LP variants in the *ENG* gene, 20 were novel, and, although not tested for all families, at least 3 were de novo variants. Similarly, among the 59 P/LP variants in the *ACVRL1* gene, 9 were novel and at least one was a de novo variant.

Table 2 shows the details of the identified pathogenic variants. In the *ENG* gene, the majority (60/80) of the pathogenic variants were private mutations unique to a single family and the variants were widely distributed in exons 1–13 coding for the 5’-UTR and the extracellular domain of endoglin. In the *ACVRL1* gene, as previously reported [3], the variants were more commonly found in exons 5–10 which encompasses the serine/threonine kinase domain. Again, the majority (25/59) of these variants were unique to a single family although those in exons 8–10 tended to be shared by multiple (2–7) families.

**Table 2:** Details of the identified pathogenic/likely pathogenic variants

Nucleotide change	Amino acid change	Exon	Previously reported?	Number of families
ENG				
c.-127C > T	decreased translation		No	1
c.67 + 1G > A	splicing defects		No	1
c.97C > T	p.Gln33*	2	Yes	1
c.125 T > G	p.Val42Gly	2	No	1
c.132_133delTA	p.Thr45Hisfs	2	No	1
c.219G > A	p.Thr73 = (skipping of exon 2)	2	No	2
c.239 T > C	p.Leu80Pro	3	No	1
c.277C > T	p.Arg93*	3	Yes	1
c.277delC	p.Arg93Glufs*9	3	No	1
c.319delC	p.Leu107Cys fs	3	No	1
c.357C > G	p.Tyr120Ilefs*42	3	No	1
c.360 + 1G > C	splicing defects		No	1
c.360 + 1G > A	splicing defects		No	3
c.433delC	p.Gln145Argfs*18	4	No	1
c.461_462insG	p.Ile156Hisfs	4	No	1
c.478delG	p.Ala160Leufs*3	4	No	1
c.494C > A	p.Pr165His	4	No	1
c.497A > C	p.Gln166Pro	4	Yes	2
c.524-1G > C	splicing defects		No	1
c.581_595del15b	p.Leu194_Pro198del5AA	5	No	1
c.586 T > A	p.Trp196Arg	5	No	1
c.611_628del18b	p.Val204_Leu209del	5	No	1
c.614G > C	p.Arg205Pro	5	Yes	1
c.647_658del	p.His215_Ala218del	5	No	1
c.685delG	p.Ala229Profs*5	5	No	1
c.760C > T	p.Gln254*	6	No	1
c.785 T > C	p.L262P	6	Yes	1
c.816 + 2 T > A	splicing defects		No	3
c.817-1G > C	splicing defects		No	1
c.937 T > C	p.Ser313Pro	7	No	1
c.944 T > A	p.Val315Glu	7	No	1
c.952-961del10b	p.Pro318Alafs	7	No	1
c.965_966delTT	p.Ile322Serfs*11	7	No	1
c.991G > A	p.Gly331Ser	7	Yes	1
c.992-1G > A	splicing defects		No	1
c.1087 T > A	p.Cys363Ser	8	Yes	1
c.1089 T > G	p.Cys363Trp	8	No	1
c.1103 T > C	p.Met368Thr	8	Yes	2
c.1109 T > C	p.Leu370Pro	8	Yes	1
c.1134G > A	p.Ala378 = (splicing defect)	8	Yes	1
c.1134 + 1G > C	splicing defects		No	1
c.1134 + 1delG	splicing defects		No	1
c.1140_1141insCTACCCAGCATTTG	p.Lys381Leufs*5	9	No	1
c.1160_1173delTGACCTTCTGGGAC	p.Leu387Profs*4	9	No	1
c.1169G > A	p.Trp390*	9	Yes	1
c.1181G > A	p.Cys394Tyr	9	Yes	1
c.1195delA	p.Arg399Glyfs*22	9	No	1
c.1209_1210insTT	p.Val404Leufs*18	9	No	1
c.1235G > A	p.Cys412Tyr	9	Yes	2
c.1268A > G	p.Asn423Ser	9	Yes	1
c.1306C > T	p.Gln436*	10	Yes	1
c.1311 + 2 T > C	splicing defects		No	3
c.1319 T > C	p.Val440Ala	11	No	1
c.1346_1347delCT	p.Ser449Phefs*51	11	No	1
c.1411C > T	p.Gln471*	11	Yes	1
c.1429-2A > G	splicing defects		No	1
c.1465C > T	p.Gln489*	12	No	1
c.1513G > T	p.Glu505*	12	Yes	1
c.1517 T > C	p.Leu506Pro	12	Yes	2
c.1649_1650insAC	p.Val551Argfs*2	12	No	1
c.1672_1684del13b	p.Gly558fs	12	No	1
c.1675_1678delTCTC	p.Ser559Lysfs*13	12	No	1
c.1687delG	p.Glu563Lys fs	13	No	1
del exons 3–8		3–8	No	1
del exons 3–8		3–8	No	1
del exons 3–14		3–14	No	1
del exons 13–14		13–14	No	1
del exons 9–14		9–14	No	1
ACVRL1				
c.90_102delGCTGGTGACCTGC	p.Leu31Argfs*19	3	No	2
c.95 T > A	p.Val32Glu	3	No	1
c.203delG	p.Gly68Alafs*54	3	No	1
c.270C > G	p.Cys90Trp	3	Yes	1
c.352C > T	p.Gln118*	4	Yes	1
c.430C > T	p.Arg144*	4	Yes	1
c.448C > T	p.Q150*	4	Yes	2
c.480_486dupCAGTCTC	p.Ile163fs	4	No	1
c.505C > T	p.Gln169*	4	Yes	1
c.525 + 1G > C	splicing defects		No	1
c.525 + 1delG	splicing defects		Yes	1
c.598C > G	p.Arg200Gly	5	Yes	3
c.614 T > G	p.Val205Gly	5	Yes	2
c.772 + 3_772 + 4dupAA	unknown, splicing defects?		No	1
c.830C > G	p.Thr277Arg	7	No	1
c.839A > G	p.His280Arg	7	Yes	2
c.899 T > C	p.Leu300Pro	7	No	1
c.926G > T	p.Gly309Val	7	Yes	1
c.956G > T	p.Gly319Val	7	No	1
c.969_970insA	p.Pro324Thr fs*73	7	No	1
c.982C > T	p.His328Tyr	7	Yes	1
c.982C > A	p.His328Asn	7	No	1
c.994A > T	p.Lys332*	7	Yes*	1
c.1044C > G	p.Asp348Glu	7	No	1
c.1069C > T	p.Gln357*	8	Yes	1
c.1120C > T	p.Arg374Trp	8	Yes	1
c.1121G > A	p.Arg374Gln	8	Yes	2
c.1132C > T	p.Pro378Ser	8	Yes	1
c.1231C > T	p.Arg411Trp	8	Yes	3
c.1232G > A	p.Arg411Gln	8	Yes	4
c.1271C > T	p.Pro424Leu	9	Yes	1
c.1327C > T	p.Cys443Arg	9	No	1
c.1345C > G	p.Pro449Ala	9	No	2
c.1412G > A	p.Cys471Tyr	10	No	2
c.1435C > T	p.Arg479*	10	Yes	7
c.1436G > A	p.Arg479Gln	10	Yes	1
c.1451G > A	p.Arg484Gln	10	Yes	3

Table 3 shows the age-specific phenotypes of all *ENG*- and *ACVRL1*-HHT patients. As the patients grow older, their chances of receiving definite/probable diagnosis increased, and, after age 40, almost all patients can be clinically diagnosed as definite HHT by the Curaçao criteria [5]. As previously reported, epistaxis was the most common symptom found in almost all patients with *ENG*- or *ACVRL1*-HHT after 12 years of age. Overall, organ or visceral AVMs were found in 84.6 and 79.3% of the patients with *ENG*- and *ACVRL1*-HHT, respectively. Pulmonary and brain AVMs were more common in *ENG*-HHT as compared with *ACVRL1*-HHT (69.1 vs. 14.4%, 34.0 vs. 5.2%) and identified earlier in life although the incidence of pulmonary AVM below 6 years of age (28.6%) was lower as compared with that above 6 years of age (68.8–100%). Hepatic AVM was more common in *ACVRL1*-HHT (31.5 vs 73.2%) and identified relatively later in life although the incidence started to rise after 6 years of age. Interestingly, in contrast with the previous reports, we observed an increased incidence of cerebral AVM (34.0% in *ENG*-HHT and 5.2% in *ACVRL1*-HHT) in our cohort as opposed to the previously reported incidence at 8–20.9% in *ENG*-HHT and 0–2.4% in *ACVRL1*-HHT [1, 4, 22, 23]. Spinal AVM was also found more commonly at 2.5% and 1.0% in *ENG*- and *ACVRL1*-HHT, respectively. Additionally, the incidence of gastric AVM (13.0% in *ENG*-HHT, 26.8% in *ACVRL1*-HHT) was higher as compared with that of duodenal AVM (3.1% in *ENG*-HHT, 5.2% in *ACVRL1*-HHT). This is in contrast with the previous studies reporting a higher incidence of intestinal AVM among those with GI-AVM [24–26].

**Table 3:** Age-related phenotypes of *ENG* and *ACVRL1*-HHT patients

	*ENG*							*ACVRL1*						
Age (years)	0–5	6–11	12–19	20–39	40–59	60-	Total (%)	0–5	6–11	12–19	20–39	40–59	60-	Total (%)
No of patients	14	16	10	43	52	27	162	3	3	6	21	39	25	97
Curaçao criteria (N = 259)														
Definite	6	11	8	38	51	27	141 (87.0)	1	1	4	15	39	25	85 (87.6)
Probable	3	4	2	5	1	0	15 (9.3)	1	1	2	4	0	0	8 (8.2)
Unlikely	5	1	0	0	0	0	6 (3.7)	1	1	0	2	0	0	4 (4.1)
Clinical presentation														
Epistaxis (N = 259)	9	14	10	43	52	28	156 (96.3)	1	2	6	19	39	25	92 (94.8)
Telangiectasia (N = 259)	1	5	8	30	45	27	116 (71.6)	0	0	3	11	39	25	78 (80.4)
AVMs of organs (N = 252)	7	13	10	35	47	25	137(84.6)	0	1	2	14	36	24	77 (79.3)
Pulmonary (N = 251)	4	11	10	32	37	18	112 (69.1)	0	1	0	5	6	2	14 (14.4)
Brain (N = 245)	5	9	5	14	12	10	55 (34.0)	0	0	1	1	3	0	5 (5.2)
Hepatic (N = 199)	0	1	2	9	20	19	51 (31.5)	0	1	2	12	34	22	71 (73.2)
Spinal (N = 102)	0	2	0	0	1	1	4 (2.5)	0	0	0	0	1	0	1 (1.0)
GI tract (N = 97)	0	0	0	1	11	12	24 (14.8)	0	0	0	0	14	12	26 (26.8)
Esophageal	0	0	0	0	0	1	1 (0.6)	0	0	0	0	1	0	1 (1.0)
Gastric	0	0	0	0	10	11	21 (13.0)	0	0	0	0	14	12	26 (26.8)
Duodenal	0	0	0	1	2	2	5 (3.1)	0	0	0	0	3	2	5 (5.2)
Colonic	0	0	0	0	2	5	7 (4.3)	0	0	0	0	3	1	4 (4.1)

For 80 *ENG*-HHT and 59 *ACVRL1*-HHT families, detailed phenotype information of multiple family members was available (Additional file 1: Table S1). In 30 (71.4%) *ENG*-HHT families, obvious intrafamilial phenotypic heterogeneity not related to the age of examination was observed. The differences were mostly manifested as the difference in the site of organ AVM between individuals. In the *ACVRL1*-HHT families, the incidence of intrafamilial phenotypic heterogeneity was lower, found in 7 (24.1%) families. The intrafamilial heterogeneity of organ AVM was found similarly across all organ AVMs.

## Discussion

In our cohort of Japanese patients, the *ENG* mutations were dominant (*ENG*/*ACVRL1* ratio at 1.35) although the difference was smaller as compared with our own previous smaller series [19]. Previous reports showed a considerable difference in the incidence of *ENG*- vs *ACVRL1*-HHT with the *ENG* dominance in the US (*ENG*/*ACVRL1* at 1.58) [27], Netherlands (3.40) [17], Denmark (4.05) [15], and Korea (1.47) [17] as opposed to the *ACVRL1* dominance in France and Italy (0.37) [28], Germany (0.89) [29], and China (0.41) [18]. The founder effects could be one of the explanations for these differences although it is unclear whether the previously identified, small-sized founder effects could account for all these differences [30].

Overall, the breakdown of mutation types in our series was similar to that of previous reports for both the *ENG* and *ACVRL1* genes (Table 1) [3, 15, 22]. Variants in the *ENG* gene were distributed throughout the gene with no obvious hot spots while mutations were more frequently found in the region spanning the serine/threonine kinase domain of the *ACVRL1* gene (Table 2). Also, as previously reported, the incidence of null mutations in the *ENG* gene was higher as compared with that of the *ACVRL1* gene [3].

Clinical features were mostly similar to those reported for other populations with an increased incidence of pulmonary and cerebral AVMs in *ENG*-HHT, and of hepatic AVM in *ACVRL1*-HHT [1, 2, 4, 19, 31]. Notable differences, however, include an increased incidence of cerebral, spinal, and gastric AVMs in our cohort.

Studies have reported a considerable difference in the incidence of various organ AVMs in those with the same causative genes [2, 3, 32]. There are also phenotypic differences even within the same families as shown in the current study. These differences likely reflect the difference in the environmental factors, ascertainment biases, or the difference in the other genetic backgrounds acting as the second hits for the development of symptoms [8]. Previous reports on the presentation of HHT in monozygotic twins showed that the presentations are similar but different supporting the influence of both genetic and environmental contribution for the development of symptoms [33–35].

The reason why the cerebral, spinal, and gastric AVMs were more common in our series is not clear. It has been reported that the incidence of cerebral hemorrhage is twofold higher in Japanese as compared with that of Caucasians [36]. Similarly, the incidence of gastric cancer is markedly increased in east Asian countries including Japan compared with that in other areas of the world [37]. The increased incidence of cerebral and gastric AVMs might have common backgrounds of these organ vulnerabilities.

## Conclusions

The largest mutational and clinical study in Japanese HHT showed an *ENG*-HHT dominance over *ACVRL1*-HHT. The mutational spectrum was similar to those reported for other populations although the cerebral, spinal, and gastric AVMs were more commonly seen.

## Supplementary Information

**Additional file 1:** Detailed mutational and clinical data of all patients.

## Data Availability

Detailed mutational and clinical data of all patients are submitted as supplemental Table 1.

## Abbreviations

HHT: Hereditary hemorrhagic telangiectasia
*ENG*: Endoglin
*ACVRL1*: Activin A receptor like type 1
*SMAD4*: SMAD Family Member 4
AVM: Arteriovenous malformation
GI: Gastrointestinal
P/LP: Pathogenic/likely pathogenic
